# A debate on working memory and cognitive control: can we learn about the treatment of substance use disorders from the neural correlates of anorexia nervosa?

**DOI:** 10.1186/s12888-016-0714-z

**Published:** 2016-01-16

**Authors:** Samantha J. Brooks

**Affiliations:** UCT Department of Psychiatry and Mental Health, Groote Schuur Hospital, Anzio Road, Observatory Cape Town, South Africa

**Keywords:** Anorexia nervosa, Substance use disorder, Working memory, Cognitive training, Neuroplasticity, MRI

## Abstract

**Background:**

Anorexia Nervosa (AN) is a debilitating, sometimes fatal eating disorder (ED) whereby restraint of appetite and emotion is concomitant with an inflexible, attention-to-detail perfectionist cognitive style and obsessive-compulsive behaviour. Intriguingly, people with AN are less likely to engage in substance use, whereas those who suffer from an ED with a bingeing component are more vulnerable to substance use disorder (SUD).

**Discussion:**

This insight into a beneficial consequence of appetite control in those with AN, which is shrouded by the many other unhealthy, excessive and deficit symptoms, may provide some clues as to how the brain could be trained to exert better, sustained control over appetitive and impulsive processes. Structural and functional brain imaging studies implicate the executive control network (ECN) and the salience network (SN) in the neuropathology of AN and SUD. Additionally, excessive employment of working memory (WM), alongside more prominent cognitive deficits may be utilised to cope with the experience of negative emotions and may account for aberrant brain function.

**Summary:**

WM enables mental rehearsal of cognitive strategies while regulating, restricting or avoiding neural responses associated with the SN. Therefore, high versus low WM capacity may be one of the factors that unites common cognitive and behavioural symptoms in those suffering from AN and SUD respectively. Furthermore, emerging evidence suggests that by evoking neural plasticity in the ECN and SN with WM training, improvements in neurocognitive function and cognitive control can be achieved. Thus, considering the neurocognitive processes of excessive appetite control and how it links to WM in AN may aid the application of adjunctive treatment for SUD.

**Electronic supplementary material:**

The online version of this article (doi:10.1186/s12888-016-0714-z) contains supplementary material, which is available to authorized users.

## Background

This debate article is written to pose the question: can a neurobiological hypothesis for excessive appetite control, derived from neurobiological data of anorexia nervosa (AN) help to direct adjunctive treatment for the control of substance use disorder (SUD)? Specifically, the role of working memory (WM) in relation to other cognitive strategies in the employment of appetite control will be considered. In order to comment on and attempt to answer this question, the following will be discussed: a) some recent research into the neuropsychological and neurobiological profile of AN; b) the neuropsychological and neurobiological profile of addiction with a focus on SUD; c) evidence regarding the neurobiology of WM; d) how WM training has been implemented to evoke neuroplasticity, clinical improvements and transferability to self-regulation skills in schizophrenia, attention deficit hyperactivity disorder (ADHD) and SUD; e) the limitations and criticisms of WM training; f) how WM training might be used as an adjunct to treatment for those with SUD, and potentially also for those with eating disorders.

Given that this article examines where WM fits in to the complex aetiology of AN and SUD, a brief description of WM and two contemporary theories, namely Global Workspace Theory (GWT) and Bayesian Probabilistic Inference (BPI) will follow, that provide suggestions as to how WM might be utilised to achieve appetite control. This will be done before briefly summarising the neuropsychological and neurobiological profiles of AN and SUD in relation to WM.

**Fig. 1:**
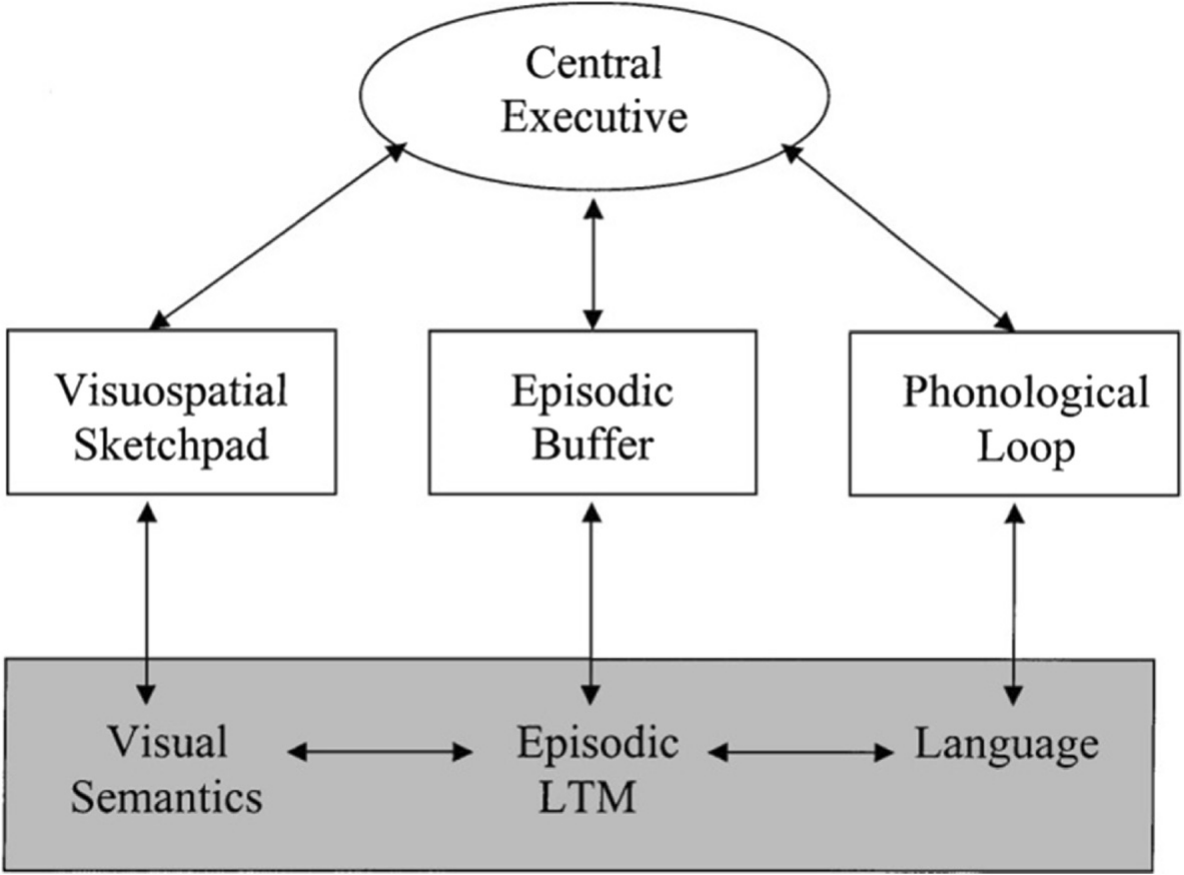
The original working memory model by Baddeley [1]. Reproduced via open access Wikimedia: https://commons.wikimedia.org/wiki/File:Working-memory-en.svg

The theoretical framework for WM was first described over 40 years ago in a book chapter by Baddeley and Hitch [1] a term synonymous, to some extent, with the limited capacity short-term memory store (see Fig. 1). However, whereas short-term memory is described as a storage process, WM rather refers to the structure and function of neural mechanisms that enable temporary storage, organisation and manipulation of memory while dynamically attending to and processing other information. Specifically, WM can be defined as “…the holding mechanism in the mind for a small amount of information that is kept in a temporarily heightened state of availability. As such, it should contain what we think of as the conscious mind, but also captures the broader role of on-going processing and temporary memory functions outside of conscious awareness.” [2]. Thus, if we take the viewpoint that WM functions to toggle between cognitive-emotion interactions in the brain, providing as a by-product a sense of conscious self-regulation, we may form a hinge model of the mind from which excessive, cognitive control and excessive engagement in appetitive processes swings. However, this viewpoint does not presuppose that WM is synonymous with consciousness or cognitive control (which introduces the infinite regress problem of homunculus management), but rather that the subjective experience of varying degrees of cognitive control, or self-regulation, automatically arises from the neural capacity to flexibly toggle between not mutually exclusive cognitive and affective states.

**Fig. 2:**
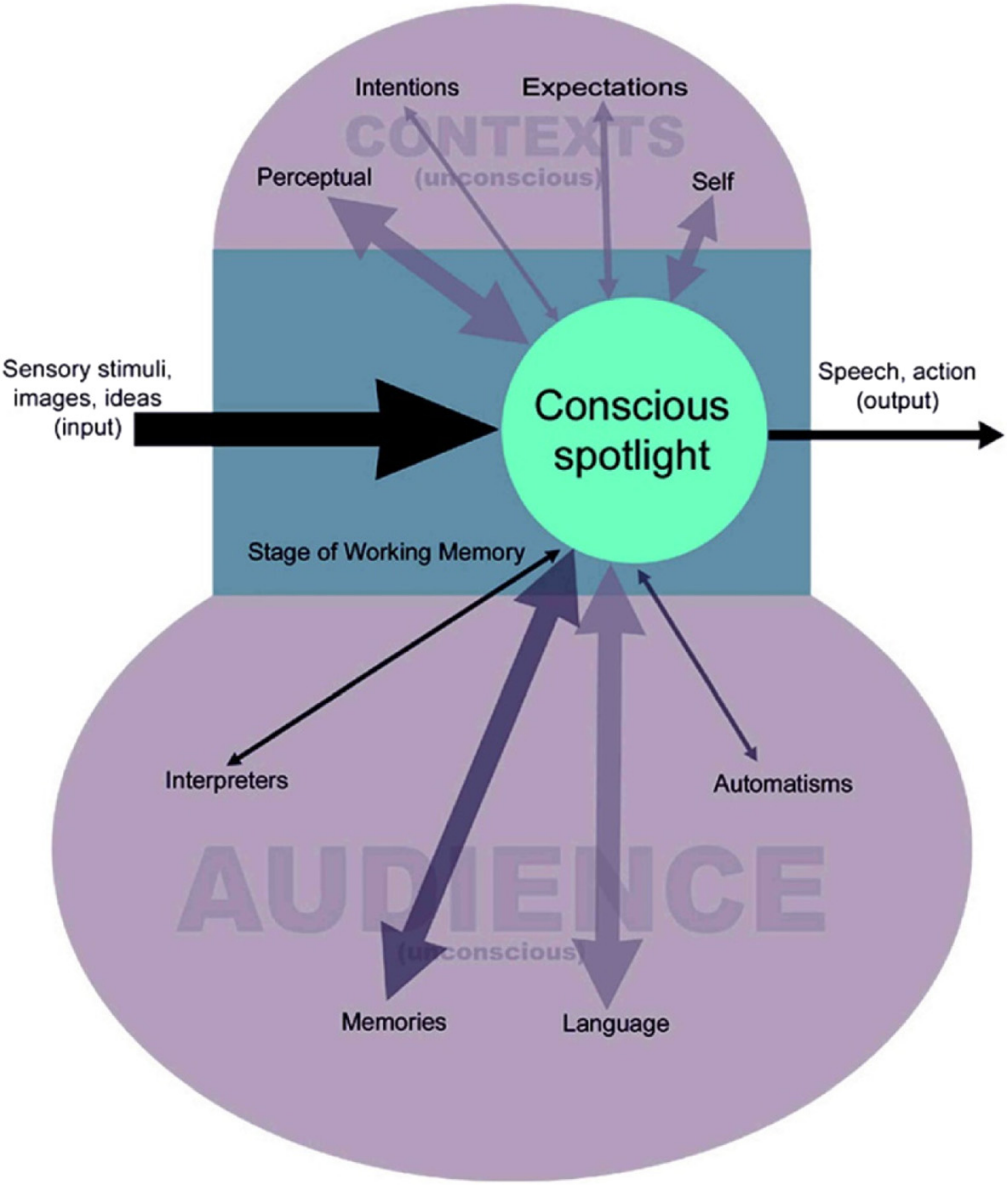
Bernie Baars’ Global Workspace Model incorporating working memory. Reproduced with permission via email communication from Professor Bernard Baars

A contemporary neurobiological theory of WM is formulated by the Global Workspace Theory (GWT, [3]), see Fig. 2. The theory posits that focal ambiguity in the brain (e.g. how attention is drawn to one of many immediate or delayed salient, rewarding possibilities) is solved by conscious precepts emerging out of unconscious, ‘backstage’ events that form a coalition within cortico-limbic tracts. The GWT helps to address the issue that the brain does not possess a controlling homunculus, but that various neural systems compete for limited conscious access at any given moment, and that the transient presence of WM is experienced consciously (and perhaps volitionally), fuelled by dynamic unconscious systems. The notion that decision-making is derived from backstage non-conscious processes as opposed to consciously and with free-will, has been shown previously by various researchers [4, 5], particularly in relation to overt action selection [6, 7]. Moreover, the underlying neural mechanisms of unconscious tendencies to act are particularly pertinent to the question posed by this article, in terms of the cognitive control over consuming food or an illicit substance. For example, if a decision to act has been determined by prior unconscious neural processes it begs two questions. How do people suffering with AN override the primary appetitive drive to consume food with sometimes-fatal rigidity? And how do individuals with SUD learn to follow complex cognitive strategies to engage in drug taking despite awareness of harmful consequences? Perhaps the answer to these questions lies in how traits and cognitive biases of people with AN and SUD contribute to styles of decision-making under conditions of uncertainty – for example, delaying a decision to act (or eat) [8] versus ‘jumping-to-conclusions’ [9] respectively.

The presence of cognitive biases relevant to the disease state (e.g. food for eating disorders, drug paraphernalia for substance dependence) suggests that conscious deliberation is perhaps not central to WM and decision-making in those with AN and SUD, but may be a by-product of the SN impinging on higher order cognitive processes. In this vein, the compulsive and complex aetiology that underlies appetite restriction in those with AN and continued substance use in those with SUD may be driven by prior sensory experience of, for example, negative emotional states. Such negative emotional states (e.g. anxiety, emotional abuse) may unconsciously bias WM processes or become resistant to updating in the presence of uncertainty (e.g. new and/or inconsistent stimulation) in the environment.

**Fig. 3:**
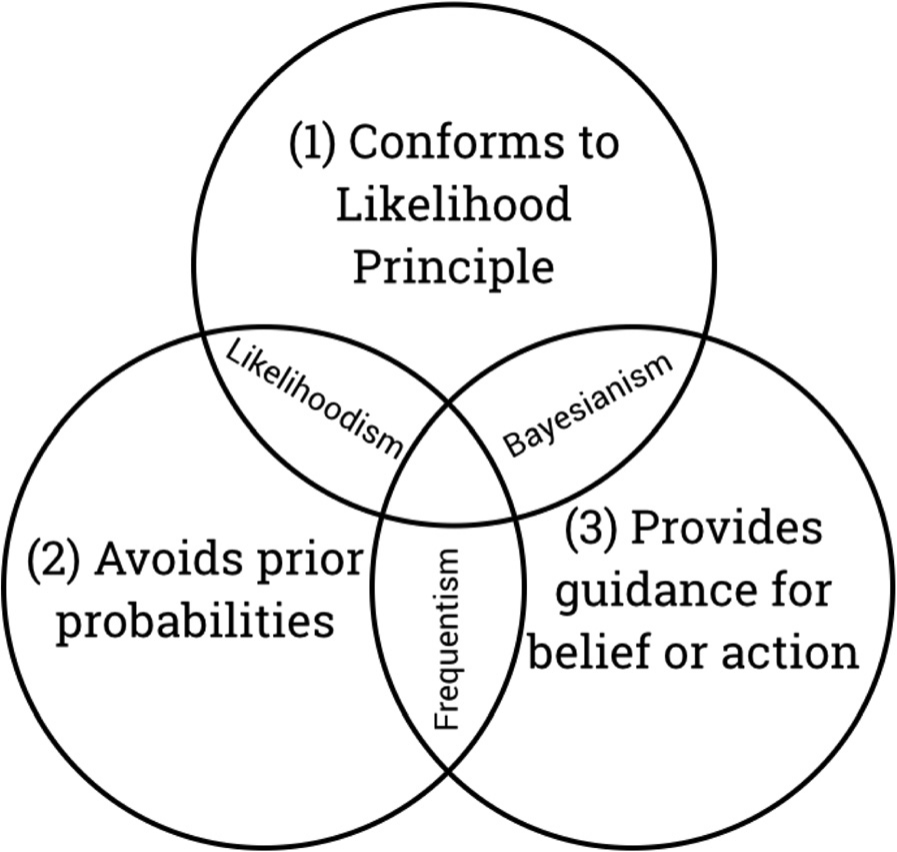
Greg Gandenberger’s Bayesian Probabilistic Inference Model to explain how Bayesianism provides guidance for belief or action, particularly under conditions of uncertainty. Reproduced with permission via email from Gregory Gandenberger(http://gandenberger.org/category/philosophy-of-science/bayesianism/). 1) Likelihoodism is based on a belief system that an outcome will occur. 2) Frequentism is based on prior experience and the probability that an event will occur; 3) Bayesianism is based on updating belief system via frequency of exposure, and informs action tendencies in the presence of uncertainty

The influence of uncertainty on the dynamic updating of WM based on prior experience is described in the theory of Bayesian Probablisitic Inference (BPI [10]) and variations in WM capacity are implicated to modulate BPI [11]. See Fig. 3. BPI provides a framework for modelling how a person integrates information from multiple cues (e.g. interoceptive, exteroceptive) and from prior knowledge about self, world and others to update perceptual inferences about probabilities in the environment [12]. For example, it could be that an increased WM capacity (e.g. inherited or learned) enables a person to hold in mind a greater number of predictions in the presence of perceived uncertainties for a longer period, which increases the probability that complex future outcomes can be predicted and prior knowledge more accurately updated. Furthermore, deliberating on a greater number of predictions and observations about uncertainty in the environment may increase the subjective states of cognitive control, self-regulation, etc. [12], and an increased WM capacity may enhance the acute subjectivity of these states.

### a) WM and the neurobiological profile of anorexia nervosa (AN)

Against this background, one could hypothesise that those with AN have increased WM capacity, due to, for example, genetic susceptibility or learning. WM capacity is likely reinforced in terms of BPI [13] by deliberating on strategies to restrict food intake amid a high availability of food (despite a level of uncertainty as to whether food restriction will be possible). It is worth noting that people with AN are shown to be intolerant of uncertainty in comparison to healthy controls and appear to gather more cues from the environment than non-eating disordered people before making a decision [14] which may also contribute to deficits in social and emotional processing [15]. Similarly, people suffering with AN learn to eat the minimum amount to stay alive and while doing so likely reinforce rigidly held unconscious prior beliefs dictating that appetite control and a thin body is synonymous with negative emotion regulation (e.g. ‘nothing tastes as good as skinny feels’ [16]) and reward [17]. In contrast to AN, those who binge eat appear to be at the opposing extreme of an impulse-control spectrum [18], characterised by a lack of self-control, a heightened reward response to food and reduced WM capacity [19, 20]. Thus, rigidly adhering to priors in order to make decisions during conditions of perceived uncertainty (e.g. whether the motivation to eat can be restricted, and whether unpredictable emotions in self and others can be effectively controlled) may contribute to excessive WM capacity and related clinical observations such as perfectionism, lack of central coherence (global thinking), inability to set-shift (adapting to flexible strategies) and social-emotional processing deficits [21].

It is hypothesised in this article that excessive, and thus dysfunctional WM is employed in those with AN. However, it is important to emphasise that WM deficits are a small part of a larger, more complex set of symptoms in those with AN. For example, the cognitive-interpersonal maintenance model of AN [21] posits that a strong attention to detail and an inability to flexibly toggle between rules (weak set shifting) are inherited vulnerabilities but that other social and familial factors exacerbate these vulnerabilities. Furthermore, people with AN have social emotion deficits including sensitivity to criticism, and deficits in emotion regulation. Thus, while WM might be integral to excessive appetite control, there are certainly other factors to consider in the complex aetiology of AN.

Other issues hampering the elucidation of the impact of WM on the symptomatology of AN include a) the assessment of WM, b) how WM is associated with ED symptoms, c) the involvement of specific brain regions and d) WM improvements after therapy (see Table 1 and below for citations). The assessment of WM in AN has to date included the N-back task (remembering letters 1,2 or 3 positions prior to a target for example); the Working Memory Index of the Wechsler Adult Intelligence Scale (WAIS); the Wechsler Memory Scale (WMS) – specifically the Digit Span backwards task; a counting span task; a task that involves remembering the position of an arrow previously presented and a spatial WM task that involves remembering the position of a lit window in a series of houses or the position of hidden blue tokens. Additionally, some studies found that distractions (e.g. negatively rated body images, subliminal and supraliminal images of food) worsen WM task performance in those with AN. In terms of ED symptoms, restricting AN has been associated with superior WM performance, coinciding with low weight status and depression. Higher maternal IQ and education level may be linked to better WM performance in children who are born to mothers with ED symptoms. Anxiety may worsen WM capacity, and a longer duration of ED illness might be linked with excessive WM capacity. However, 4 out of 11 studies also found no correlation between ED symptoms and WM ability. Only 3 brain imaging studies to date have examined neural responses during WM tasks in those with AN, revealing increased bilateral dorsolateral prefrontal cortex (DLPFC), premotor cortex, left middle temporal gyrus and right precuneus activation. Additionally, those who have recovered from AN may have greater amygdala and fusiform activation, and greater suppression of the medial prefrontal cortex during a WM task when viewing images of bodies that have been rated negatively. In sum, most studies show superior [20, 22–26], some deficit [27, 28] and others show no difference [29–31] in WM performance in AN compared to binge eaters or otherwise healthy people.

**Table 1:** Chronological list of studies examining working memory in people with AN

Author (chronological) and reported WM effect in AN	Participants	Type of WM task	Brain imaging	Main findings	ED symptoms and WM findings
Israel et al. (2015) [20] BETTER	Female adults with ED: ED-R (n = 19) ED-BP (n = 27) Limitation: no control group, although the authors’ highlight that this is a pilot study.	N-back task with variable cognitive load (arithmetic) and stress (positive and negative feedback)	fMRI (1.5 T)	ED-R performed consistently better than the ED-BP group on all N-back versions. Further, the ED-R group had increased right DLPFC and premotor cortex activation during the 2-back vs. 0-back task in comparison to ED-BP. ED-BP had weaker WM activation than ED-R.	Binge ED symptoms are associated with worse WM performance whereas restricting ED symptoms are associated with better WM performance. Right posterior prefrontal cortex activation is weaker during WM in those with binge ED. Age, BMI, Education influenced these findings, whereas anti-depressant medication and chronicity of illness did not.
Weider et al. (2015) [28] WORSE	Female adults with ED: AN (n = 40) BN (n = 39) HC (n = 40)	Working Memory Index (WAIS-III Manual): Paced Auditory Serial Addition Test (PASAT) 3, PASAT 2, WAIS-III (Letter Number Sequencing, Digit Span), WMS-R (Spatial Span)	None	The AN group had lower WM scores than both BN and HC.	Lowest lifetime BMI and depressive symptoms explained the worse WM performance in the BN group but not the AN group.
Lao-Kaim et al. (2014) [31] NO DIFFERENCE	Female adults with ED: AN (n = 31) HC (n = 31)	N-back task (0, 1, 2 and 3 back). The authors’ specifically examine verbal WM, incorporating the phonological loop, the phonological store, sub-vocal rehearsal and the central executive.	fMRI (1.5 T)	No significant difference in WM task performance. All groups showed increased activation in the bilateral IPL, bilateral middle and superior frontal gyri extending into the DLPFC, left precuneus and right insula. The AN group additionally showed positive trends in the left middle temporal gyrus, right precuneus and left IFG.	Duration of illness may be associated with lower WM accuracy in the AN group. However, anxiety and depression scores were not shown to influence WM ability in AN.
Kothari et al. (2013) [26] BETTER	Male and female 10 yr old children of mothers with ED (n = 6192)	The counting span task - a computer-based task that simultaneously assesses the processing and storage components of WM.	None.	Increased WM capacity in children with mothers of AN compared to those whose mothers did not have a history of AN (e.g. those with BN or non-ED mothers).	Higher maternal education level and child IQ level may mediate the effect of better WM scores.
Brooks et al. (2012) [24] BETTER	Female adults: R-AN (n = 13) HC (n = 20)	The N-back task (1-back and 2-back), presented on a computer screen, with additional subliminal images of food, neutral and aversive scenes	None	Females with R-AN were significantly better at the N-back task (fewer total errors), compared to HC. However, their superior performance on the N-back task was compromised only when subliminal images of food were presented. This suggests that subcortical (e.g. non-conscious) processing of food stimuli interfered with WM capacity.	Higher levels of anxiety correlated with number of errors during the WM task in those with R-AN.
Pruis et al. (2012) [25] NO DIFFERENCE	Female adults: Recovered AN (n = 15) HC (n = 16)	Memoranda arrow was shown, followed by a distracting image of a body (negative, neutral, positive or scrambled), and then another arrow. Participants were instructed to indicate whether the second arrow presentation was in the same orientation as the first.	fMRI (3 T)	HC and recovered AN wormen did not differ on the WM task overall. Additionally, body images that were rated as negative were more disruptive to WM processes in both groups, but presentation of other distracting body images had no effect on WM. Amygdala and fusiform activation were greater in women who had recovered from AN than in controls when viewing images of bodies during the WM task. There were no group differences in DLPFC activity. However, there was more suppression of medial prefrontal cortex activity in women who had recovered from AN in comparison to controls when negatively rated images were presented during the working memory task.	More years recovered may have had an influence on negative ratings of bodies and activation levels in the amygdala.
Nikendei et al. (2011) [30] NO DIFFERENCE	Female adults: R-AN (n = 34) BP-AN (n = 19) WS-AN (n = 16) HC (n = 30)	Wechsler Memory Scale Revised (WMS-R) – Digit span backwards.	None.	Currently ill and weight-restored AN patients did not differ significantly from healthy controls with respect to WM. However, there was impaired mmediate and delayed verbal recall performance in acute AN patients that was found irrespective of AN subtype, and that persisted in weight-restored AN patients.	ED symptoms or comorbidities did not correlate with WM performance.
Hatch et al. (2010) [23] BETTER	Female adolescents AN (n = 37) HC (n = 45)	IntegNeuro-computerized Battery using the N-back continuous performance test of sustained attention.	None.	During underweight status, AN patients had superior WM capacity in comparison to HC.	ED symptoms or comorbidities did not correlate with WM performance.
Dickson et al. (2008) [22] BETTER	Female adults: R-AN(n = 24) HC (n = 24)	The N-back task (1-back and 2-back), presented on a computer screen, with additional subliminal and supraliminal images of food, neutral and aversive scenes	None.	Participants with AN had superior WM performance compared to the HC during the subliminal condition, but were more distracted than HC by the supraliminal condition.	Duration of illness correlated positively with number of errors made by the AN group.
Fowler et al. (2006) [29] NO DIFFERENCE	Female adults: AN (n = 25) HC (n = 25)	Cambridge Neuropsychological Test Automated Battery (CANTAB). Spatial WM is a test of spatial working memory and strategy performance to find individually hidden ‘blue tokens’ without returning to a box where one has previously been found	None.	No impairments were observed in spatial WM.	ED symptoms or comorbidities did not correlate with WM performance.
Seed et al. (2002) [27] WORSE	Female adults: AN (n = 20) HC (n = 20)	Spatial WM as part of a test battery. A picture of a house is presented for 5 s. The house has nine windows, four of which are lit. A series of 36 presentations of the same house in which just one window is lit follows, and the participant has to respond ‘Yes’ if the window was one of the four lit in the original presentation, or ‘No’ if it was not. Cortisol measures were also taken.	None.	WM performance was impaired in females with AN compared to HC, but cortisol levels did not differ between groups.	ED symptoms or comorbidities did not correlate with WM performance.

*WM* working memory, *AN* anorexia nervosa, *BN* bulimia nervosa, *ED-R* eating disorder restricting type, *ED-BP* eating disorder binge-purge type, *BMI* Body Mass Index, *IQ* Intelligence Quotient, *fMRI* functional magnetic resonance imaging, *DLPFC* dorsolateral prefrontal cortex, *IPL* inferior parietal lobe, *IFG* inferior frontal gyrus, *R-AN* restricting anorexia nervosa, *BP-AN* binge-purge restricting anorexia nervosa; 5 studies reported BETTER WM performance in AN compared to HC; 2 studies reported WORSE WM peformance and 4 studies reported NO DIFFERENCE. 3 studies to date have examined neural function in relation to WM performance in those with AN

The heterogeneity of findings across studies of WM in AN could also be due, in part, to the transdiagnostic nature of symptoms both between (e.g. traits) and within (e.g. states) individuals with ED [18]. Nevertheless, based on current research, there are three main points as to why the neurobiology of WM capacity is an emerging platform upon which to examine the mechanism of appetite and impulse control. First, WM is regarded as a cognitive mechanism that is shown to exert control over distracting arousing stimulation [32]. In line with this, WM and not other cognitions, such as response-inhibition, have been shown to interact with non-consciously processed appetitive images of food in those with AN [23]. Second, WM function is associated with fronto-parietal cortex activation [33–35], brain regions that have been shown be most susceptible to neurobiological changes in those with AN [32, 36–40]. And third, neuropsychological impairments in those with AN (e.g. executive dysfunction, deficits in somatic and emotion processing, rigid thinking, perseveration) appear to be characteristic of increased function in the DLPFC, anterior cingulate cortex (ACC), striatum and parietal cortex, regions of the executive control network (ECN) and salience network (SN), which provide clues as to the neural mechanisms of appetite control [37, 39, 40] and some further pointers towards the involvement of WM.

Neurobiological similarities and differences between AN, bulimia nervosa (BN) and addiction are also found [46, 70]. For example, a behavioural economic approach to how rewarding substances (e.g. food, drugs) may annex normal learning systems in the brain is at odds with the traditional model of distinct disease processes and treatments [71]. However, in the light of this article it is pertinent to consider similarities and differences in neural systems across disorders such as AN and SUD, given that variations in cognitive control of appetite can help to uncover the neural relationship between conscious tertiary control cognitions and primary process appetitive systems [19] that may be associated with WM function. For example, Kaye and colleagues suggest that extra-ordinary activation of the ECN (which is associated with WM), while diminishing optimal cognitive functioning, aids the suppression of appetite and consummatory behaviour associated with the SN and acts as a protective factor to prevent those with AN from developing SUD [46]. From another perspective, negative emotions that are difficult to consciously attend to might be dealt with by excessive activation of the ECN in those with AN.

**Fig. 4:**
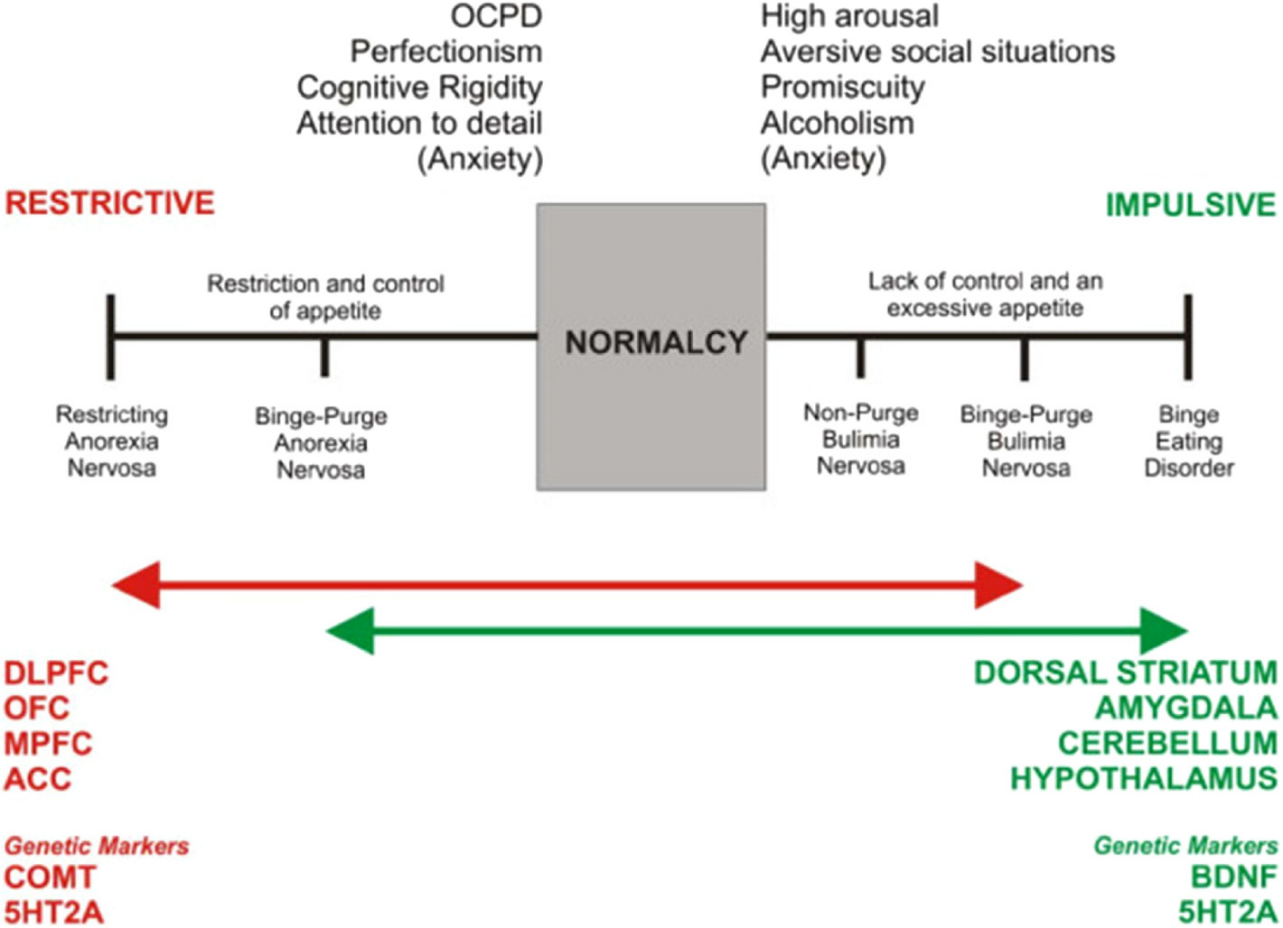
Neurobiological impulse-control model of temperamental dominance in ED [18]OCPD = Obsessive-Compulsive Personality Disorder; DLPFC = dorsolateral prefrontal cortex; OFC = orbitofrontal cortex; MPFC = medial prefrontal cortex; ACC = anterior cingulate cortex; COMT = catechol-o-methyltransferase; BDNF = Brain Derived Neurotrophic Factor; 5HT2A = 5-Hydroxy-Tryptophan-2A

In line with this view, Kaye and colleagues suggest that those with BN and SUD learn to self-medicate in response to the experience of negative emotion by consuming, in an uncontrolled and impulsive/compulsive manner, large quantities of rewarding substances and experiences (e.g. food, drugs, alcohol, sex). Bingeing on or excessive regulation of rewarding sensations has the effect of temporarily reducing negative emotion in the absence of optimal self-regulation in conjunction with dysregulation of the serotonergic system [72, 73] particularly involving the frontotemporal cortex [74]. Conversely to AN and healthy people, those who binge eat, like those with SUD have reduced activation of prefrontal cortex networks, combined with an increased activation in the limbic system [19, 64, 75]. Thus, it could be, in line with an impulse-control spectrum neural model [18], and a behavioural economic model [52] that those with AN learn to excessively recruit the ECN via WM function, which diminishes optimal cognitive performance and the reward value of food, whereas those with BN and SUD have weakened WM and learn to consume rewarding substances as a means to saturate the conscious experience of negative emotion (See Fig. 4).

**Fig. 5:**
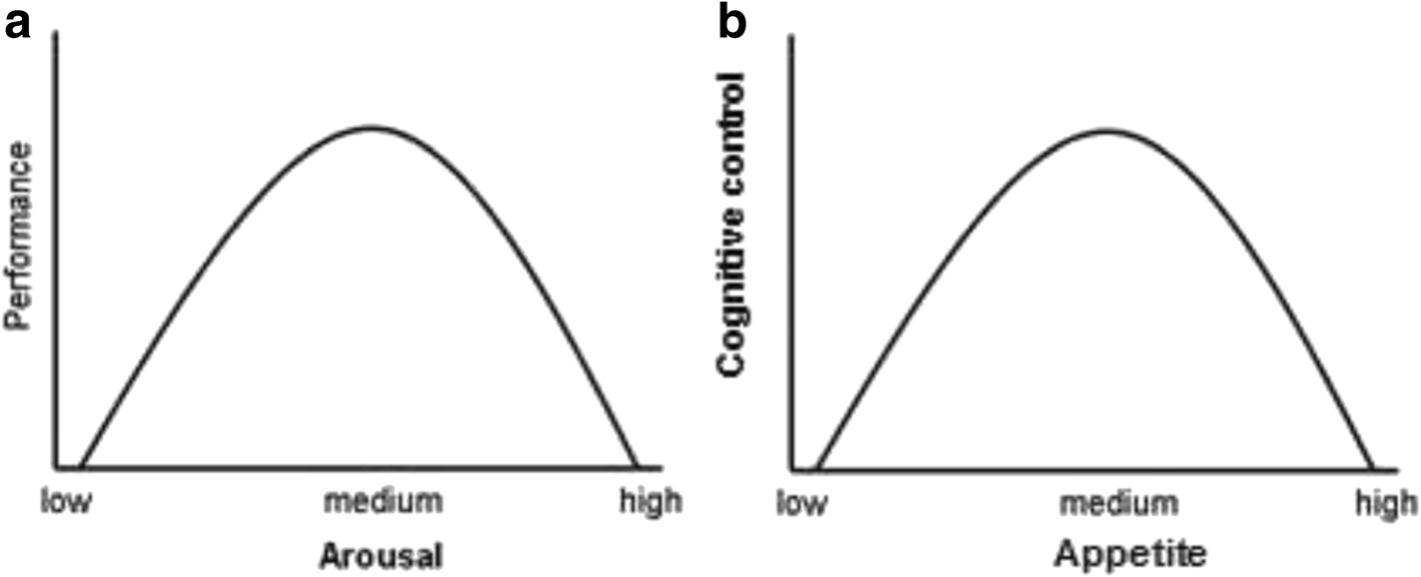
A model of cognitive control of appetite. **a** The original Yerkes-Dodson Law [76], showing that optimal performance occurs when medium arousal is present, but that both low and high arousal can be detrimental to performance. Reproduced via open access Wikimedia: https://commons.wikimedia.org/wiki/File:Yerkes-Dodson_wet.png; **b**) Updated model to depict cognitive control of appetite, applicable to restricting anorexia nervosa and addictive behaviours such as SUD and binge eating. Low appetitive processes (e.g. due to satiation with substance/food or hijacked by negative emotion such as anxiety or anger) coincide with low cognitive control. High appetitive processes (e.g. reward sensitivity, real or perceived) coincide with low cognitive control because either reward responses impinge on executive functioning or executive functioning is overloaded. Optimal cognitive control (e.g. self-regulation and effective cognitive-emotion neural interactions) is suggested to occur when appetitive drive is within the medium range

Another model of AN has recently emerged that is akin to regarding habitual appetite restraint as a reward reinforcement, or an addiction, whereby excessive recruitment of ECN is considered not to dampen reward processes, but rather to hijack the mesolimbic reward system and increase the saliency of disease-related cues [17]. This model fits well with recent empirical evidence of increased activation of top-down control and bottom-up reward processes, and heightened cognitive bias in response to salient food stimuli in those with AN [67, 68]. The view that excessive appetite restraint is an addiction in itself also fits well with the model being proposed in this article. Specifically, it is suggested at the foundation of the model that an optimal level of cognitive control of appetite enables effective cognitive-emotion processing and self-regulation. However, extreme or limited activation of the ECN can present as addictive symptomatology, that is to say, lack of cognitive control over compulsions (see Fig. 5). This model is in line with the Yerkes-Dodson Law of arousal and performance, in that both extreme low and high levels of arousal – similarly, high and low levels of appetite control - can lead to sub-optimal cognitive control and performance [76].

### b) WM and the neurobiological profile of addiction

Addiction encompasses a variety of behavioural disorders such as SUD (illicit substances, alcohol, nicotine), pathological gambling, Internet use disorder and sexual dysfunction and reflects altered reward processing [77], pain and negative emotion susceptibility [78, 79]; hijacked decision-making and lack of self-control [71]. Parallels have been drawn between addiction and eating disorders with a binge-eating component in terms of deficits in self-regulatory control over consummatory behaviours [19], and the influence of dopamine signalling during impulsive decision-making [80]. Addictive behaviours appear to stem from an inability to cope with intrusive negative cognitions and emotions (e.g. painful past experiences, lack of immediate reward/tension release, fear of loss), which are either consciously and deliberately suppressed via engagement in rewarding experiences, or unconsciously repressed via the avoidance of a negative experience [81]. In a recent meta-analysis, chronic substance abuse was associated with deficits in verbal WM [82] and sustained abstinence from drug taking is associated with improvements in WM that are concomitant with prefrontal cortex alteration [83]. However, as with eating disorders, it is not clear whether WM deficits are due to developmental vulnerability or to current illness effects.

Deficits in WM in those with SUD before treatment are linked to sub-optimal self-regulation and higher rates of risky decision-making [84–86] and are in line with a dual system model incorporating activation of deliberate top-down cognitive control versus automatic bottom-up reward and arousal [87]. Specifically, behavioural, cognitive and affective deficits include apathy, disinhibition and executive dysfunction, lack of response inhibition, mental inflexibility, poor WM and suboptimal processing of affective stimuli [88]. Furthermore, attention bias to addiction-salient stimuli appears to be associated with automatic cognitive processes and deficits in WM [89]. Abstinence from an addictive substance is also associated with poor WM performance that can be improved with cognitive training intervention [90, 91]. Thus, addiction research suggests that optimal response selection, WM and attention, which perhaps collectively enable cognitive control and emotion regulation, are most susceptible during illness, due to a failure of response inhibition that is observed as impulsive and compulsive acts in those with SUD [92].

### c) Evidence regarding the neurobiology of WM and links to appetite/impulse control in AN and SUD

**Fig. 6:**
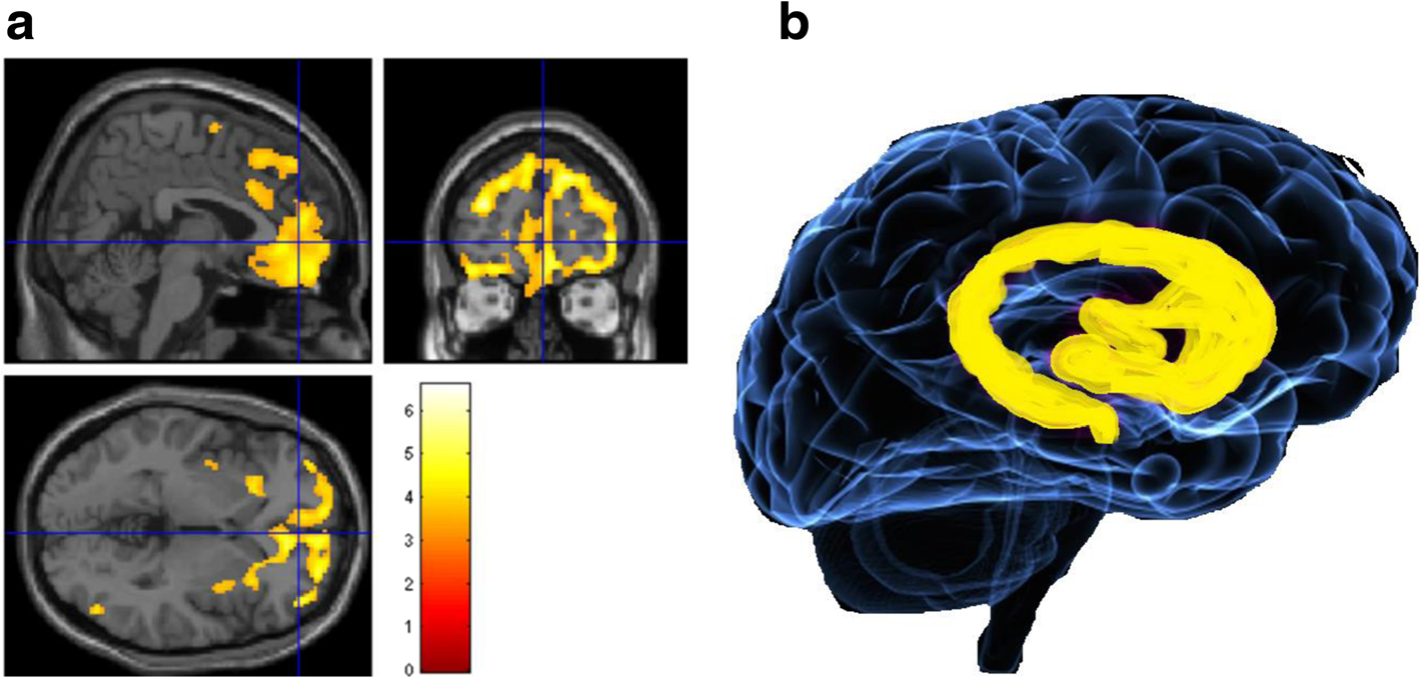
Brain regions most implicated in addiction. **a** Prefrontal cortex volume is shown to be reduced in those with SUD, produced via unpublished data with permission from Dr Samantha Brooks; **b**) A cartoon of the basal ganglia, associated with arousal, motivation, primary process affective states. Together, the prefrontal cortex and areas of the basal ganglia form the cortico-striatal pathway, which is implicated in the neuropathology of SUD and AN

WM is a candidate mechanism for exploring the neural bases of cognitive control of appetite and impulsivity given that WM deficits are concomitant with aberrant fronto-parietal network structure and function in both AN and SUD. For an illustration of susceptible brain regions see Fig. 6 and Additional file 1. Additionally, the GWT and BPI help to provide some potential insights into how WM processes contribute to excessive versus deficit cognitive control in those with AN and SUD respectively. Addictive behaviour in itself could be a reflection of how uncertainties are perceived to be incongruent with existing prior beliefs or expectations, to the extent where prediction errors, and therefore a heightened subjectivity of a lack of cognitive control, are increased when a rewarding goal is consistently sought. In line with this suggestion, at the beginning of their illness, people with SUD tend to engage in goal-directed drug seeking behaviour that reinforces reward expectations about consuming the drug [103], which has parallels with excessive recruitment of ECN and WM in those with AN [46]. However, as SUD progresses, goal-directed behaviour transfers from delayed to habitual behaviour, driven by immediate rewards, particularly for those who have a neurobiological vulnerability for impulsive behaviour [104]. This shift from goal-directed to habitual behaviour in those with chronic SUD could reflect a reasoning style that is shaped by a reduction in prediction error and a consistent congruence with priors based on a high degree of reinforcement in the presence of environmental uncertainty (e.g. whether the drug can be sought and ingested). It must also be remembered that ingestion of the drug damages corticostriatal pathways (See Fig. 6) to the extent that suboptimal WM capacity likely ensues in those with SUD. This could indicate, conversely to those with AN where existing priors are rigidly fixed, that as SUD progresses the uncertainties in the environment flood, rather than reinforce and update prior expectations.

In terms of the neurobiology of WM, GWT and BPI, regions of the lateral prefrontal cortex, ACC, parietal cortex, striatum and hippocampus are linked to deliberative processing, reduced delay discounting and the experience of self-control for the suppression of incongruent impulses [32, 59, 87]. Non-consciously processed salient stimuli activate the amygdala, bilateral ACC, bilateral insular cortex, hippocampus and primary visual cortex [105], and are limbic regions associated with affective states that influence decision-making processes. These regions are implicated in the pathophysiology of AN and SUD as described above, and could be an indication that both disorders share common neural mediators for the dysregulation of negative emotion, especially when derived from childhood or adolescence [106]. The prefrontal cortex is vital to aid planning ahead with multiple steps held in WM, as described by temporal attention framing, which can allow rewards not to be selected, or to be deferred [13, 87, 107–109]. Additionally, BPI highlights the role of reinforcement learning in the brain and implicates the corticostriatal pathways in reward prediction [110], involving differential activation in this pathway for immediate versus delayed rewards [107]. Furthermore, when prediction error is present and priors are updated based on new information, there appears to be greater ACC and striatum activation [106], and increased ACC activation is sometimes observed in both AN [70] and SUD [111].

## Discussion

Against the background of the neurobiology of AN, SUD and neural mechanisms of WM, a brief discussion will follow as to how WM training may be implemented to promote neuroplasticity and the experience of cognitive control in other psychiatric conditions. Furthermore, some of the existing constraints to WM training will be highlighted, ending by discussing some future directions as to how WM training might be used as an adjunct to standard evidence-based treatment for both SUD and AN, particularly those who have been resistant to treatment.

### d) How WM training has been successfully implemented to evoke neuroplasticity, clinical improvements and transferability to other skills in schizophrenia, attention deficit hyperactivity disorder (ADHD) and SUD

Emerging evidence, particularly in those with schizophrenia, attention deficit hyperactivity disorder (ADHD) and SUD, shows that targeted intensive cognitive training can induce normalcy and greater efficiency within neural system operations, while potentially fostering inherent plasticity processes [112]. In those with schizophrenia, repetitive cognitive training has successfully targeted cognitive deficits in perception, WM, attention, and social cognition, or combinations of these functions [113]. Further, among other clinical factors, ‘brain reserve’ measures may predict better outcome following cognitive training [113]. Brain imaging studies of those with schizophrenia undergoing cognitive training reveal structural and functional changes in key prefrontal brain regions associated with WM, executive functioning and cognitive inhibition [112, 114, 115]. It is pertinent to consider WM improvements in schizophrenia in the light of WM training for SUD, given that protracted SUD can provoke the onset of psychosis, suggesting that similar neural systems are at state [116].

Recent studies of those with ADHD, which can also present as comorbidity with SUD [117], have demonstrated clinically significant effects of WM training, particularly in lowering inattention and impulsivity [118–120]. Additionally, learning difficulties in those with ADHD are improved with WM training and sustained for up to 8 months [121]. The current leader in WM training for ADHD is CogMed™, a computer-based WM package that claims to increase WM capacity over 5 weeks of progressively difficult levels, 5 times per week at home, work or school, using a variety of WM tasks tailored to school children or adults (http://www.cogmed.com/cogmed-training-method). The CogMed™ package includes games that utilise already existing WM tasks such as digit span, spatial awareness, verbal fluency, rule-changes and attention to detail. CogMed™ has been shown to improve WM function and non-trained response inhibition and reasoning skills in other cognitive domains [122], and is linked to parent-reported lower inattention and impulsivity rates in children [123], in children born with low birth weight [124] and in children with low language ability [125]. Overall, CogMed™ has generated research over the last decade that appears to foster benefits in visuo-spatial and verbal WM that transfer to better attention at 6 months [122, 126]. Further, improvements on impulsivity and attention measures following WM training are related to fronto-parietal and striatal functional alterations in children with ADHD [123, 124]. However, there is also some criticism of CogMed™ in that WM training may only provide near transfer effects, and it is not known whether these effects are durable to other cognitive skills that do not mimic the tasks included in the CogMed™ package [127–130].

In those with SUD, computerized WM training has been shown to reduce impulsivity and delay discounting (lowering the preference for immediate over delayed rewards) among stimulant users [131]. However, unlike other fields where WM training is being effectively utilised, WM training research for SUD is in its infancy, and it is currently unclear whether training in those with SUD should be reserved for those with demonstrable cognitive deficits [132], and which cognitive targets (WM or others) result in the largest clinical effect size. Nevertheless, a contemporary behavioural economic theory underlying the use of WM training for those with SUD is that rewarding substances annex learning mechanisms in the brain, resulting in a dysregulation of value systems and distortion in decision-making that can be strengthened with training [71]. More specifically, it is suggested that WM training can help to strengthen a dual-system of deliberative over automatic neural responses to reward [87]. In line with a dual-system neural model of SUD, the mesolimbic reward system, including the nucleus accumbens and ventromedial prefrontal cortex support automatic responses to rewarding stimuli, whereas the lateral prefrontal and parietal cortices (regions associated with WM) are more closely linked to deliberation and the exertion of self-control in the suppression of impulses [59, 87].

### e) Constraints/limitations to the WM model of cognitive training for SUD

The most common hurdle for WM training is to demonstrate that improvements to cognitive performance are long-term, transferable to other non-trained cognitive skills and better than other clinical interventions. Some researchers using CogMed™ have not demonstrated long-term, transferable effects that are significantly better than other clinical methods [133], whereas others have [122, 126]. Similarly, WM training in those with SUD appears to reduce impulsivity, but it is not clear how long this effect lasts [120]. Another issue is whether it is more beneficial to train using multiple executive function domains and WM tasks or to focus on neural functioning using less, and simpler tasks to evoke plasticity within a less widespread, measurable domain [134]. Furthermore, it must be noted that using adjunctive WM training might be best implemented in those who have been resistant to standard evidence-based treatment.

Using a WM model to explain cognitive control of appetite and impulsivity is also fraught with pitfalls. WM is only one domain of executive functioning, and the ability to regulate complex cognitive-affective interactions most likely involves many other neural functions. Furthermore, while some of the research into the influence of non-conscious processes on cognitive-affective interactions has been discussed above, it is still hotly debated as to how to measure non-conscious processes that are highly inter- and intra-individually variable. Moreover, the perspective of BPI on WM as a mechanism for the subjective experience of cognitive control in AN and SUD remains difficult to interpret, although it seems to raise important questions as to the problem of appetite and impulse control. For example, for a person suffering with AN, do priors become rigidly fixed and impenetrable to prediction error and normal learning processes? For a person suffering with SUD, do prior expectations become overwhelmingly shaped by uncertainty, or does the experience of drug taking also rigidly shape prior expectations about the world? Another issue is the difficulty of honing in on the neural correlates of appetite and impulse control, and of automatic versus deliberative dual systems, due to the heterogeneity of methods, the transdiagnostic nature of illness and the lack of clear knowledge concerning how neural systems dynamically interact over the course of illness.

### f) Future directions: How WM training can be used as an adjunct to treatment for those with SUD, and potentially also for those with eating disorders who are resistant to standard evidence-based treatment

To aid the progression of cognitive training research, several principles for achieving robust and specific integration of neural representations have been proposed [135]. Firstly, a desired skill must be practiced via repetitive training on a progressive and frequent schedule (e.g. between 4-8 weeks for half an hour per day). Additionally, ‘scaffolding’ should occur whereby accurate performance on first level easier trials should become gradually more difficult, while maintaining a trial-by-trial reward. Further, attention and reward systems must be consistently engaged with a high proportion of the learning trials attended to, performed correctly and rewarded. Finally, cognitive training approaches should support generalization of improved function to real world environments, such as learning-driven improvements in cognitive and socio-affective operations to untrained stimuli, tasks, and, most importantly, to cognitively demanding real-world situations. Despite these recommendations however, research into WM training is in its infancy, and is therefore perhaps best reserved for those patients who continue to be resistant to standard evidence-based treatment.

In line with recommendations for optimal WM training, researchers in Cape Town, South Africa have developed a Smartphone App called ‘Curb Your Addiction (C-Ya)’ that has evoked improvements in impulse control in patients with methamphetamine dependence (Brooks et al., under review). During this preliminary investigation brain-imaging data has revealed structural and functional differences in the fronto-striatal circuitry following 4 weeks of WM training. The research in Cape Town is being progressed by examining whether out-patients and other psychiatric cohorts have similar beneficial neurobiological alterations following WM training. In terms of AN or other EDs, there have been no studies to date to examine whether WM training improves flexibility and/or relaxes cognitive control over eating. It may at first appear counter-intuitive to consider that WM training can improve cognitive performance in those with AN, given that they already have a rigid control over appetite, and that it may be more suitable to strengthen control for those who engage in binge eating. However, if one considers that extra-ordinary appetite restriction could in itself be an addictive process that is associated with a range of cognitive deficits, WM training may improve the ability to toggle flexibly between cognitive and affective states. Nevertheless, more research is needed in both ED and SUD to determine whether WM training, as an adjunct to boost standard evidence-based treatment, improves brain structure and function in domains associated with the cognitive control of appetite and impulsivity.

## Conclusions

There is a phenomenological quality to mind, consciously experienced when WM is exerted to assume control, albeit temporarily, over non-consciously derived impulses and appetites. Simply put, a person suffering from anorexia nervosa is sometimes conscious of compulsively over-exerting self-control despite the risk of debilitating and/or fatal consequences. Similarly, a person with SUD is sporadically aware that negative emotions are only temporarily substituted by the compulsive consumption of a rewarding substance. It is thus intriguing to consider whether WM training can extend the temporary conscious experience of cognitive control via a process of neural plasticity to improve the clinical prognosis for those with AN and SUD. Has this commentary article answered the question as to whether the neuroscience of extra-ordinary cognitive control in those with anorexia nervosa can be used to determine the usefulness of WM training as an adjunct to the treatment of SUDs? Yes and no. Yes, it has provided a précis as to how WM might be utilised in those with anorexia nervosa to suppress appetite, and how it may be annexed in those with SUD. Yes, it has provided suggestions based on evidence as to how WM training can evoke neural plasticity. No, it has not shown that the temporary and limited conscious WM capacity can be expanded, or that potentially beneficial effects of WM training remain in clinical populations. No, it has not fully clarified how WM is used to regulate appetite, although it has discussed some of the theories as to how WM might function in the brain. Thus, it would be beneficial for further research to be conducted in future to consider the role of WM in the conscious experience of appetite and impulse control.

## Additional file

Additional file 1:
**Other neurobiological eating disorder research.** Other neurobiological SUD research [41–45, 47–51, 53–58, 60–63, 65, 66, 69, 93–102]. (DOCX 16 kb)

## Abbreviations

ACC: anterior cingulate cortex
ADHD: attention deficit hyperactivity disorder
AN: anorexia nervosa
ASD: autistic spectrum disorder
BN: bulimia nervosa
BPI: bayesian probablilistic inference
C-Ya: Curb Your Addiction
DLPFC: dorsolateral prefrontal cortex
ECN: executive control network
ED: eating disorder
fMRI: functional magnetic resonance imaging
GWT: Global Workspace Theory
HC: healthy control
OCD: obsessive-compulsive disorder
OCPD: Obsessive-Compulsive Personality Disorder
OFC: orbitofrontal cortex
SN: salience network
SUD: substance use disorder
WM: working memory
