# Supplementary material for: A debate on working memory and cognitive control: can we learn about the treatment of substance use disorders from the neural correlates of anorexia nervosa?
Source: BMC Psychiatry. 2016 Jan 16;16:10. doi: 10.1186/s12888-016-0714-z (PMC4715338; doi:10.1186/s12888-016-0714-z)
Supplement: Additional file 1: — Other neurobiological eating disorder research. Other neurobiological SUD research [41–45, 47–51, 53–58, 60–63, 65, 66, 69, 93–102]. (DOCX 16 kb) [file 12888_2016_714_MOESM1_ESM.docx]

**Other neurobiological eating disorder research**

Prefrontal and parietal cortex function is associated with WM ability [41] and obsessive-compulsivity in those with AN [36,39], which are also regions of the ECN. It is hypothesised in this article that WM supports ruminating on cognitive strategies associated with the ECN, which ultimately regulates activation of the SN. Contemporary brain imaging studies suggest that there is reduced resting state activation in the SN in those with EDs, particularly those currently ill and recovered from AN [42,43] although others suggest increased SN activation may also contribute to hyperactivity [44]. Recent reviews and meta-analyses have provided further comprehensive summaries of neurobiological differences in AN that contribute to the neuropsychological profile as described above and the excessive, often fatal control of appetite [38, 45-47]. The reviews highlight reduction in grey and white matter in the anorectic versus the healthy state [48,38]. Regional grey matter differences in the ECN and SN are observed in patients with AN compared to healthy individuals, such as reduced ACC and orbitofrontal cortex (OFC) volume [49-52]; reduced limbic and somatosensory cortex volume, namely the amygdala, hippocampus, striatum, insula and parietal lobe [53-59]. Some preliminary evidence suggests that protracted cognitive restraint over a period of approximately ten years of illness with AN may be linked to increased volume in the right DLPFC [58] a brain region associated with WM function and impulse control [59].

Functional differences between AN and healthy individuals are observed with advances in functional Magnetic Resonance Imaging (fMRI), using paradigms that include passive responses to disease-salient stimuli, actively thinking about disease-salient stimuli and responses to cognitive tasks. Passively viewing images of high versus low calorie drinks is associated with activation of regions involved in reward and emotional processing, including increased activation in left insula, ACC and amygdala-hippocampal regions and decreased activation in the posterior cingulate cortex in patients currently ill with AN [60-62]. Conversely, thinking about eating food shown in images compared to non-food items activates regions associated with ECN, such as the DLPFC, medial prefrontal cortex, parietal lobe, cerebellum and visual cortex in restricting AN compared to healthy women [63-67]. This suggests that following an instruction to think about eating food, as opposed to passively attending to images of food appears mainly to activate the ECN in those with AN.

Various cognitive tasks have been employed in fMRI studies to test the neural correlates of executive dysfunction in AN, such as set-shifting, response inhibition and WM, and these tasks demonstrate differences in fronto-striatal and fronto-parietal networks that are associated with WM and may underlie a perseverative, rigid and obsessive-compulsive cognitive style [40, 39, 20]. Furthermore, fMRI findings of those with AN might reflect a greater cognitive bias for disease-salient stimuli [68], which may be driven by non-conscious activation of reward-related limbic processes (e.g. in the striatum) that interfere with cognitions such as WM [24]. In line with this, it has been shown that non-conscious dopaminergic modulation via the basal ganglia influences visual and prefrontal cortex cognitions such as selective attention, vigilance, set-shifting and executive control [69] and that emotions can exert distracting effects on WM [32]. Thus, while alterations in brain function are associated with various cognitive deficits, WM also appears to be an important factor in the complex aetiology of AN.

**Other neurobiological SUD research**

Impulsive and compulsive acts are common in those with SUD and impulse-control disorders, and are associated with differential brain structure and function in cortico-striatal circuitry [77, 93]. In those with stimulant dependence for example, aberrant structure and function in the cortico-striatal circuitry is associated with deficits in self-control, cognitive flexibility and maladaptive decision-making [94,95]. Similarly, it has been shown that dysfunction within brain systems incorporating the DLPFC, OFC and ACC are linked to behavioural, emotional and cognitive deficits in those with SUD [88]. Furthermore, the clinical observation that denial and self-deception plays a large part in the pathology of addiction may contribute to a lack of insight, executive dysfunction and reduced prefrontal cortex structure that is a cause or a consequence of SUD [96]. Finally, reduced response inhibition and reduced neural function in the default mode network (linked to internalised thoughts), which includes the temporal lobe, medial prefrontal cortex and posterior cingulate, is associated with worse WM ability [97].

Protracted abstinence in those in treatment for stimulant use can have deleterious effects on brain regions associated with emotion regulation and the processing of the physiological state of the body, such as the insula, amygdala, posterior cingulate and hippocampus [98], perhaps as a result of neurotoxicity or tonic versus phasic dopamine firing [80]. Furthermore, SUD is concomitant with neurotoxicity via aberrant dopamine release and larger volume in the mesolimbic reward circuitry, particularly in the striatum, reduced prefrontal cortex, insula, hippocampus and temporal cortex, with heightened craving associated with smaller volumes in some of these regions [99]. However, substituting substance use for a prosocial reward can have beneficial effects [100,101], especially in substance abusers with high dopamine D2/D3 receptor availability in the mesocorticolimbic pathway [102], although D2/D3 receptor availability does not appear to be related to craving [99]. The subjective experience of craving may rather be determined by a complex interplay between brain regions associated with reward sensitivity, with the processing of the current physiological state of the body and the conscious experience of cognitive control. Thus, structural and functional abnormalities in the dopaminergic corticostriatal pathway may contribute to the neuropsychological deficits observed in those with SUD, particularly in terms of craving, reward sensitivity and executive dysfunction such as WM deficits (See Fig. 6).
